# Milde COVID-19-Verläufe bei Mitarbeitenden einer Universitätsklinik: Die „erste Welle“ am Universitätsklinikum Hamburg-Eppendorf

**DOI:** 10.1007/s00103-021-03396-9

**Published:** 2021-08-10

**Authors:** Johann von Felden, Thomas Theo Brehm, Julian Schulze Zur Wiesch, Marylyn M. Addo, Ansgar W. Lohse, Johannes K.‑M. Knobloch, Till Koch

**Affiliations:** 1 I. Medizinische Klinik und Poliklinik, Universitätsklinikum Hamburg-Eppendorf, Martinistr. 52, 20246 Hamburg, Deutschland; 2 Standort Hamburg-Lübeck-Borstel-Riems, Hamburg, Lübeck, Borstel, Deutsches Zentrum für Infektionsforschung (DZIF), Hamburg, Deutschland; 3 Institut für Medizinische Mikrobiologie, Virologie und Hygiene, Universitätsklinikum Hamburg-Eppendorf, Hamburg, Deutschland

**Keywords:** COVID-19, SARS-CoV‑2, Gesundheitsarbeiter*innen, Krankheitsverlauf, Infektion, COVID-19, SARS-CoV‑2, Healthcare workers, Course of disease, Infections

## Abstract

**Hintergrund:**

Mitarbeitende in Gesundheitseinrichtungen sind direkt exponiert und damit besonders gefährdet in der anhaltenden COVID-19-Pandemie. Trotz diverser Berichte zu SARS-CoV-2-Infektionszahlen unter Mitarbeitenden deutscher Kliniken sind die Verläufe von COVID-19 bei dieser besonderen Population wenig beschrieben.

**Fragestellung:**

In diesem Kurzbeitrag sollen die Merkmale und Verläufe von Infektionsfällen unter Mitarbeitenden am Universitätsklinikum Hamburg-Eppendorf in der ersten Welle der Pandemie dargestellt werden.

**Methoden:**

Im Zeitraum 01.07.–28.07.2020 wurden 67 vormals positiv auf SARS-CoV‑2 getestete Mitarbeitende via E‑Mail eingeladen, in einem anonymen Onlinefragebogen detaillierte Angaben zum Krankheitsverlauf zu machen; 39 Personen nahmen teil.

**Ergebnisse:**

Die Teilnehmenden (58 %) waren überwiegend ≤ 39 Jahre alt (64 %) und weiblich (70 %). Die meisten berichteten über direkten Patientenkontakt (85 %), inklusive SARS-CoV-2-positiver Patient*innen (62 %). Die Beschwerden hielten im Median 19 Tage an. 85 % berichteten Fatigue, 67 % Geruchs- oder Geschmacksstörungen, 64 % Husten, 62 % Kopfschmerzen und 51 % Kurzatmigkeit. Die Verläufe waren überwiegend mild; 5 % wurden stationär behandelt. 38 % berichteten mehr als 4 Wochen nach Symptombeginn über anhaltende Beschwerden, insbesondere Geruchs- oder Geschmacksstörungen, Fatigue oder Kurzatmigkeit. Diese hatten häufiger Vorerkrankungen berichtet (53 % vs. 12 %, *p* = 0,010) und im Speziellen einen arteriellen Hypertonus (27 % vs. 4 %, *p* = 0,062).

**Diskussion:**

COVID-19-erkrankte Gesundheitsarbeitende berichteten trotz regelhafter Kontakte zu SARS-CoV-2-infizierten Patienten zum größten Teil über milde Verläufe. Ein Teil gab allerdings auch nach Monaten noch Symptome an.

## Abstract (en)

**Background:**

Healthcare workers are among the most exposed and potentially most threatened populations of the ongoing COVID-19 pandemic. Despite some reports on numbers of infections with SARS-CoV‑2 in German healthcare workers, the courses of their clinical presentation when affected by COVID-19 are not well described.

**Objective:**

In this contribution, characteristics and progressions of infected cases among healthcare workers at the University Medical Center Hamburg-Eppendorf during the first wave of the COVID-19 pandemic will be presented.

**Methods:**

Between 1 July and 28 July 2020, 67 healthcare workers, who previously tested positive for SARS-CoV‑2 via PCR, were invited via E‑mail to participate in an anonymous online questionnaire; 39 persons participated.

**Results:**

Participants (58%) were mostly ≤ 39 years old (64%) and female (70%). Most healthcare workers were involved in direct patient management (85%), including contact with SARS-CoV‑2 positive patients (62%). All participants reported acute symptoms with a median duration of 19 days. The most frequent symptoms were fatigue (85%), anosmia (67%), cough (64%), headache (62%), and shortness of breath (51%). The disease course was mostly mild with low admission rates (5%). Ongoing symptoms lasting more than four weeks post-symptom-onset, particularly anosmia, fatigue, and shortness of breath, were reported by 38%. This group more frequently had pre-existing conditions (53% vs. 12%, *p* = 0.010), specifically hypertension (27% vs. 4%, *p* = 0.062).

**Discussion:**

Healthcare workers reported mostly mild courses of COVID-19 despite increased contact with SARS-CoV-2 patients. However, some reported persistent symptoms months after infection.

## Einleitung

Mitarbeitende in Gesundheitseinrichtungen zählen zu den direkt exponierten und damit potenziell besonders gefährdeten Populationen in der COVID-19-Pandemie. Laut Robert Koch-Institut hatten sich bis zum 25.11.2020 rund 27.129 Mitarbeitende in Gesundheitseinrichtungen nachweislich mit SARS-CoV‑2 infiziert [1]. Die Übertragung von SARS-CoV‑2, dem COVID-19 auslösenden Virus, geschieht vor allem durch infektiöse Tröpfchen und Aerosole [2]. Durch adäquate Schutzmaßnahmen kann eine Infektion von Gesundheitsarbeiter*innen durch Patient*innen in der Regel verhindert werden. Gerade zu Beginn der COVID-19-Pandemie in Deutschland, im März und April 2020, waren die ergriffenen Schutzmaßnahmen in Krankenhäusern jedoch zum Teil ungenügend [3], sodass unter Mitarbeitenden die Sorge vor einer Infektion durch SARS-CoV‑2 groß war. Gründe hierfür waren zum Teil vorhandener Mangel an adäquater persönlicher Schutzausrüstung, fehlende Schulung des Personals und ein zunächst restriktiver Einsatz von Polymerase-Kettenreaktion(PCR)-Testungen aufgrund geringerer Testkapazitäten. Die Frage ob Mitarbeitende in Gesundheitseinrichtungen, die sich mit SARS-CoV‑2 infizieren, ein im Vergleich zur Gesamtbevölkerung höheres Risiko für einen schweren Verlauf von COVID-19 haben (zum Beispiel aufgrund von erhöhten Expositionsdosen), ist nach wie vor unklar. Von März bis Mai 2020 kam es an mehreren großen Kliniken zu vereinzelten Infektionen von Mitarbeitenden [4, 5]. Das Universitätsklinikum Hamburg-Eppendorf (UKE) ist ein Klinikum der Maximalversorgung mit 13.560 Mitarbeitenden, in dem pro Jahr 106.000 stationäre und 405.000 ambulante Patient*innen versorgt werden. Wir beschreiben hier die Merkmale und den zeitlichen Verlauf von Infektionsfällen mit SARS-CoV‑2, welche im Frühjahr 2020 bei Mitarbeitenden des UKE auftraten.

## Methoden

Im Rahmen der „ersten Welle“ wurden am UKE bis zum 30.06.2020 67 Mitarbeitende positiv auf SARS-CoV‑2 getestet; Testungen und Nachverfolgung wurden durch die Abteilung für Krankenhaushygiene durchgeführt. Zwischen dem 01.07. und 28.07.2020 wurden die positiv getesteten Mitarbeitenden via E‑Mail eingeladen, einen anonymen REDCap(Research-Electronic-Data-Capture)-basierten Onlinefragebogen auszufüllen. In Einklang mit der Deklaration von Helsinki wurden in Absprache mit der Hamburger Ethikkommission und den Betriebsräten aufgrund der geringen Fallzahl besondere Vorkehrungen zum Datenschutz getroffen, um eine Identifizierung der Teilnehmer*innen auszuschließen. So wurden bestimmte Datenpunkte, wie z. B. das Alter, gruppiert abgefragt. Die Ansprache der Mitarbeitenden samt Versendung des Einladungslinks erfolgte durch das Institut für Krankenhaushygiene im Rahmen einer routinemäßigen Kontaktaufnahme zur Nachsorge.

## Ergebnisse

**Figure 1:**
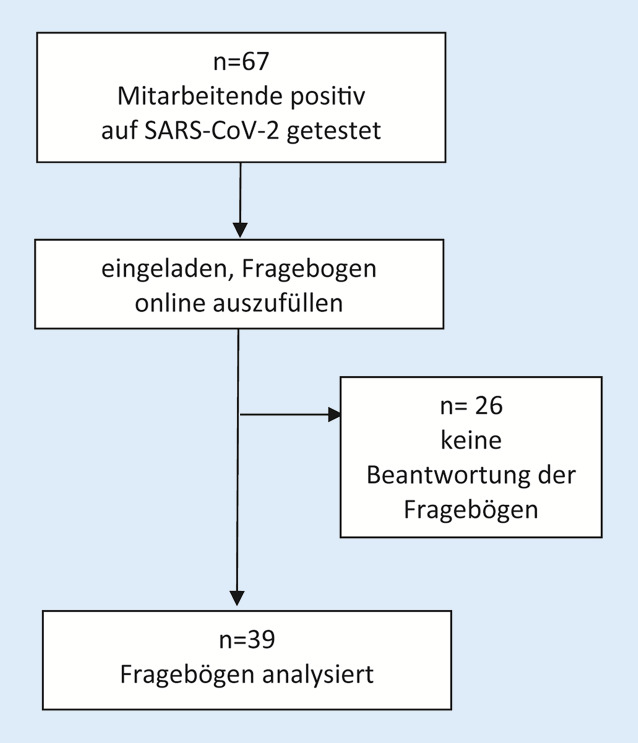

Von 67 kontaktierten Mitarbeitenden des UKE füllten 39 den Fragebogen aus (58 %; Abb. 1). Die meisten Mitarbeitenden waren jünger als 30 Jahre (*n* = 13, 33 %) oder 30 bis 39 Jahre alt (*n* = 12, 31 %). Frauen überwogen in der Studienpopulation (*n* = 27, 70 %), darunter eine Schwangere. 17 Teilnehmende (44 %) waren Pfleger*innen, 11 (28 %) waren Ärzt*innen und weitere 11 Beschäftigte (28 %) waren in anderen Bereichen eingesetzt. Zum überwiegenden Teil hatten die infizierten Mitarbeitenden direkten Kontakt zu Patient*innen (*n* = 33, 85 %), inklusive direkten Kontakts zu SARS-CoV-2-positiven Patient*innen (*n* = 24, 62 %) und Einsatzes auf COVID-19-Stationen (*n* = 15, 38 %). 20 (51 %) Mitarbeitende waren der Meinung, sich bei Patient*innen angesteckt zu haben. Details zur Studienpopulation finden sich in Tab. 1.

**Table 1:** 

	Teilnehmende (*n* = 39)
*Altersgruppe, in Jahren*	
≤ 29	13 (33)
30–39	12 (31)
40–49	1 (3)
50–59	9 (23)
≥ 60	4 (10)
*Geschlecht*	
Weiblich	27 (70)
Männlich	12 (31)
*Beruf*	
Pfleger*in	17 (44)
Ärzt*in	11 (28)
Physiotherapeut*in	2 (5)
Medizinische Fachangestellte*r	1 (3)
Andere	8 (21)
*Vermutlich angesteckt bei …*	
Patient*in	20 (51)
Kolleg*in	1 (3)
Zu Hause/privates Umfeld	6 (15)
*Vorerkrankungen*	10 (27)
*Rauchen*	4 (10)
*Medikamente*^*a*^	10 (26)
*Reise in Risikogebiete*	4 (10)

Datenpunkte sind als absolute Zahlen (Prozent) dargestellt

^a^inklusive oraler Kontrazeptiva

Alle Betroffenen hatten akute Beschwerden mit mindestens einem typischen Symptom zum Zeitpunkt der positiven SARS-CoV-2-PCR-Testungen. Die Symptome bestanden im Median für 19 Tage, wobei die Symptomdauer mit einer Spannbreite von 1 bis 129 Tagen sehr variabel war (Tab. 2). Dabei berichteten 9 Betroffene (23 %) von anhaltenden Symptomen zum Zeitpunkt der Befragung (im Median 85 Tage nach Beginn der Symptome), 15 (38 %) Betroffene berichteten von Beschwerden über eine Dauer von mindestens 4 Wochen (Tab. 2). Zu den häufigsten Beschwerden zu mindestens einem Zeitpunkt zählten Fatigue (85 %), Geruchs- oder Geschmacksstörungen (67 %), Husten (64 %), Kopfschmerzen (62 %), Kurzatmigkeit (51 %) und Myalgien (49 %; Tab. 3). Fieber ≥ 38,5 °C wurde von 15 Teilnehmenden (38 %) berichtet. 12 Betroffene (31 %) entwickelten weder Husten noch Fieber.

**Table 2:** 

	Teilnehmende (*n* = 39)
Krankheitsverlauf	
*Art der medizinischen Versorgung*	
Ambulant	37 (95 %)
Stationär	2 (5 %)
Sauerstoffgabe	0
Intensivstation	0
Mediane Symptomdauer	19 Tage (Spannweite 1–129)
Teilnehmende mit Symptomen	39 (100 %)
Teilnehmende mit Symptomen 4 Wochen PSO	15 (38 %)
Teilnehmende mit Symptomen zum Zeitpunkt der Befragung	9 (23 %)

*PSO* Post Symptom Onset, nach Symptombeginn

**Table 3:** 

	Teilnehmende (*n* = 39)
Symptome zu mindestens einem Zeitpunkt	*N* (%)
Husten	25 (64)
Schnupfen	15 (38)
Heiserkeit oder Halsschmerzen	17 (44)
Fatigue	33 (85)
Kopfschmerzen	24 (62)
Muskelschmerzen (Myalgien)	19 (49)
Gelenkschmerzen (Arthralgien)	8 (21)
Erhöhte Temperaturen (< 38,5 °C)	6 (15)
Fieber (≥ 38,5 °C)	15 (38)
Nachtschweiß	15 (38)
Schüttelfrost	13 (33)
Kurzatmigkeit (subjektiv)	20 (51)
Luftnot (bei Belastung)	5 (13)
Durchfall (Diarrhö)	7 (18)
Übelkeit	4 (10)
Erbrechen	2 (5)
Geruchs- oder Geschmacksstörungen	*26 (67)*

**Figure 2:**
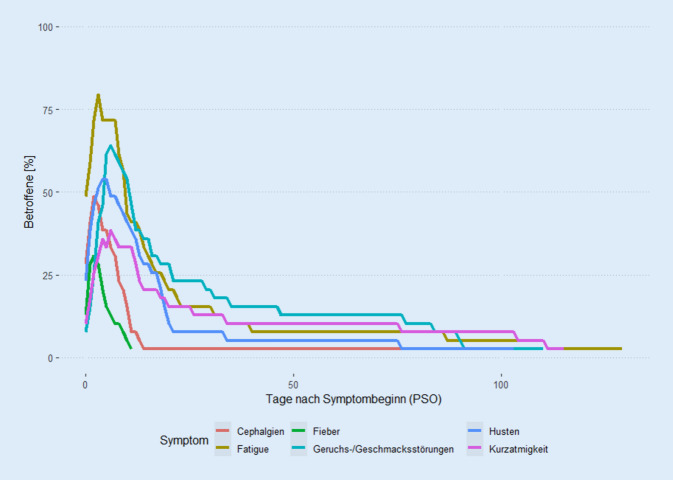

Die Verläufe waren überwiegend mild, es gab lediglich 2 Mitarbeitende die stationär behandelt wurden, jedoch keine Aufenthalte auf der Intensivstation (Tab. 2). 15 Teilnehmende (38 %) berichteten mehr als 4 Wochen nach Symptombeginn (Post Symptom Onset [PSO]) über anhaltende Beschwerden, insbesondere über Geruchs- oder Geschmacksstörungen (*n* = 8), Fatigue (*n* = 6) und Kurzatmigkeit (*n* = 5). Diese hatten häufiger Vorerkrankungen berichtet (53 % vs. 12 %, *p* = 0,010) und im Speziellen einen arteriellen Hypertonus (27 % vs. 4 %, *p* = 0,062). Unterschiede im Verteilungsmuster der initialen Symptome zwischen Mitarbeitenden mit oder ohne lang anhaltende Symptome zeigten sich nicht. Details zum zeitlichen Verlauf der 5 häufigsten Symptome und Fieber finden sich in Abb. 2.

## Diskussion

Im Vergleich zur Gesamtbevölkerung war die Population der erkrankten Mitarbeitenden jünger, häufiger weiblich und seltener vorerkrankt, was gut zu den milden Verläufen der COVID-19-Erkrankungen passt [6]. Ob die von einigen Teilnehmenden berichteten lang anhaltenden Symptome als Ausdruck eines Long-COVID-Syndroms [7] zu werten sind und somit auch arbeitsmedizinische Relevanz haben, lässt sich erst nach einem längeren Nachbeobachtungszeitraum beurteilen. Zum Zeitpunkt des Studiendesigns (Frühjahr 2020) lagen kaum Berichte zu Langzeitfolgen einer SARS-CoV-2-Infektion vor, sodass unser Fragebogen keine detaillierten Aussagen zu langfristigen Folgen auf die Lebensqualität oder z. B. zu chronischer Fatigue treffen kann.

Weitere Limitationen dieser Studie sind die geringe Größe der Stichprobe, ein Reportingbias für schwere Verläufe/Todesfälle, ein Selection-Bias zugunsten symptomatischer Verläufe sowie der anonymisierte Charakter der Daten und die damit fehlende Möglichkeit einer Nachbefragung. Aufgrund des retrospektiven Charakters der Studie war dieses Design jedoch zur Wahrung der Anonymität der Teilnehmenden erforderlich. Insgesamt zeigte sich, dass Mitarbeitende im Krankenhaus bei eigener Infektion zum größten Teil einen milden Verlauf der COVID-19-Erkrankungen hatten, aber bei einer Minderheit lang anhaltende Symptome bestanden.

## Fazit

In einer Gruppe von Mitarbeitenden einer deutschen Universitätsklinik, die sich bis zum 30.06.2020 mit SARS-CoV‑2 infizierten, fanden sich überwiegend milde klinische Verläufe, bei einigen wenigen Individuen hielten Symptome jedoch Monate an. Ob es sich in diesen Fällen um Long-COVID-Syndrome handeln könnte und ob dies eine arbeitsmedizinische Relevanz haben wird, gilt es, weiter zu erforschen.
