# Erratum zu: Milde COVID-19-Verläufe bei Mitarbeitenden einer Universitätsklinik: Die „erste Welle“ am Universitätsklinikum Hamburg-Eppendorf

**DOI:** 10.1007/s00103-021-03406-w

**Published:** 2021-09-16

**Authors:** Johann von Felden, Thomas Theo Brehm, Julian Schulze zur Wiesch, Marylyn M. Addo, Ansgar W. Lohse, Johannes K.‑M. Knobloch, Till Koch

**Affiliations:** 1 I. Medizinische Klinik und Poliklinik, Universitätsklinikum Hamburg-Eppendorf, Martinistr. 52, 20246 Hamburg, Deutschland; 2 Standort Hamburg-Lübeck-Borstel-Riems, Hamburg, Lübeck, Borstel, Deutsches Zentrum für Infektionsforschung (DZIF), Hamburg, Deutschland; 3 Institut für Medizinische Mikrobiologie, Virologie und Hygiene, Universitätsklinikum Hamburg-Eppendorf, Hamburg, Deutschland

**Erratum zu:**

**Bundesgesundheitsbl 2021**

10.1007/s00103-021-03396-9

In der ursprünglichen Originalfassung des Artikels wurden einige Namen der Autorinnen und Autoren nicht korrekt wiedergegeben.

Die Namen wurden nun korrigiert.

